# Juvenile dermatomyositis in Western Switzerland

**DOI:** 10.1186/1546-0096-9-S1-P52

**Published:** 2011-09-14

**Authors:** F Bellutti Enders, M Hofer

**Affiliations:** 1 Pediatric rheumatology Romande, DMCP, CHUV, Lausanne and HUG, Genève, Switzerland

## Introduction

Juvenile dermatomyositis (JDM) is a rare chronic, autoimmune vasculopathic disease with mainly muscle and skin involvement. The incidence described in USA is 2.5-4.1 cases per million children; the disease is slightly more common in girls. Recent studies describe that two third of the patients followed a chronic disease course, whereas only one third showed monocyclic disease course.

## Objectives

Assess disease characteristics and outcome of JDM in Western Switzerland.

## Methods

Retrospective review of charts from 1997-2010 in pediatric rheumatology in Lausanne and Geneva.

## Results

We describe a population of 13 patients presenting a JDM followed in the two centers of pediatric rheumatology in western Switzerland from 1997-2010. The incidence of JDM in our region is similar to other studies (3.5 cases per million children). The mean age of onset was 6 years (3 - 10 years); no gender predominance was found. The mean follow up was 6 years (3 months - 14 years). All patients presented a rash (Gottron’s rash, heliotrope rash or extensor surface rash, in one patient only during follow-up), muscle weakness (assessed by CMAS and or Kendall score) and evidence of myositis (biopsy, elevated muscle enzymes, magnetic resonance imaging or by electromyography). The median delay of diagnosis was 2 months, 3 patients had a longer delay because of misleading symptoms. Follow up of more than one year is available in 12 patients. In comparison with other studies, evolution was predominantly monocyclic (50% of all cases showed remission within 2 years after diagnosis). All patients were treated by corticosteroids and Methotrexate. If skin manifestation predominated, Hydroxychloroquin was added (40% of all cases). In the six other patients with chronic polycyclic, intermittent and chronic disease course, multiple treatment changes were required including Ciclosporine, Azathioprin, immunoglobulines, Infliximab, Adalimumab, Rituximab. Only one patient had pulmonary complications (interstitial pneumonia) and 1 patient still presents functional impairment. Calcinosis was present in 3 patients (23%). All patients showed at one point impregnation of corticosteroid treatment. One patient presented pancreatitis and fungal infection related to treatment. Growth impairment was seen in 3 patients (23%).

## Conclusion

In our study we show a better outcome compared to other recent reports. This could be due to early diagnosis and rapid treatment onset or less severe presentation of the disease in our patients. Early referral to a reference center and rapid treatment with corticosteroids combined with Methotrexate may improve the outcome.

